# Development of Glycyrrhizinic Acid-Based Lipid Nanoparticle (LNP-GA) as An Adjuvant That Improves the Immune Response to Porcine Epidemic Diarrhea Virus Spike Recombinant Protein

**DOI:** 10.3390/v16030431

**Published:** 2024-03-11

**Authors:** José Bryan García-Cambrón, José Luis Cerriteño-Sánchez, Rocío Lara-Romero, David Quintanar-Guerrero, Gerardo Blancas-Flores, Brenda L. Sánchez-Gaytán, Irma Herrera-Camacho, Julieta Sandra Cuevas-Romero

**Affiliations:** 1 Programa de Doctorado en Biología Experimental, Universidad Autónoma Metropolitana, Iztapalapa, Ciudad de México 09089, Mexico; tlcbioexp@gmail.com; 2 Instituto Nacional de Investigaciones Forestales Agrícolas y Pecuarias, Centro Nacional de Investigación Disciplinaria en Salud Animal e Inocuidad, Cuajimalpa, Ciudad de México 05110, Mexico; 3 Programa de Estancia Posdoctoral, Facultad de Medicina Veterinaria y Zootecnia, Universidad Nacional Autónoma de México, Ciudad de México 04510, Mexico; chio_lrp@yahoo.com.mx; 4 División de Estudios de Posgrado (Tecnología Farmacéutica), Facultad de Estudios Superiores, Universidad Nacional Autónoma de México, Cuautitlán Izcalli, Estado de México 54740, Mexico; quintana@unam.mx; 5 Laboratorio de Farmacología, División de Ciencias Biológicas y de la Salud, Universidad Autónoma Metropolitana, Iztapalapa, Ciudad de México 09089, Mexico; gera@xanum.uam.mx; 6 Centro de Química ICUAP, Laboratorio de Bioinorgánica Aplicada, Benemérita Universidad Autónoma de Puebla, Puebla 72592, Mexico; brenda.sanchez@viep.com.mx; 7 Centro de Química ICUAP, Laboratorio de Bioquímica y Biología Molecular, Edificio IC7, Benemérita Universidad Autónoma de Puebla, Puebla 72592, Mexico; irma.herrera@correo.buap.mx

**Keywords:** porcine epidemic diarrhea virus, lipid nanoparticle, recombinant protein, immune response

## Abstract

Porcine epidemic diarrhea virus (PEDV) has affected the pork industry worldwide and during outbreaks the mortality of piglets has reached 100%. Lipid nanocarriers are commonly used in the development of immunostimulatory particles due to their biocompatibility and slow-release delivery properties. In this study, we developed a lipid nanoparticle (LNP) complex based on glycyrrhizinic acid (GA) and tested its efficacy as an adjuvant in mice immunized with the recombinant N-terminal domain (NTD) of porcine epidemic diarrhea virus (PEDV) spike (S) protein (rNTD-S). The dispersion stability analysis (Z-potential −27.6 mV) confirmed the size and charge stability of the LNP-GA, demonstrating that the particles were homogeneously dispersed and strongly anionic, which favors nanoparticles binding with the rNTD-S protein, which showed a slightly positive charge (2.11 mV) by in silico analysis. TEM image of LNP-GA revealed nanostructures with a spherical-bilayer lipid vesicle (~100 nm). The immunogenicity of the LNP-GA-rNTD-S complex induced an efficient humoral response 14 days after the first immunization (*p* < 0.05) as well as an influence on the cellular immune response by decreasing serum TNF-α and IL-1β concentrations, which was associated with an anti-inflammatory effect.

## 1. Introduction

Porcine epidemic diarrhea virus (PEDV), a member of the Alphacoronavirus family, causes an important viral disease characterized by acute diarrhea, vomiting, dehydration, and high mortality in neonatal piglets, leading to significant economic losses in the swine industry [1]. The virus genome size is 28 kb approximately, it contains two non-coding regions (UTR) located at the 5′ and 3′ terminals, four structural proteins: spike (S), membrane (M), envelope (E), and nucleocapsid (N) proteins as well as ORF1a and ORF1b encoding 14–16 non-structural proteins, and ORF3 encodes an accessory protein [2,3]. The PEDV S protein plays an important role in pathogenesis, mainly in the interaction between the virus and cell membrane (via the S1 subunit) and subsequent virus internalization in the cell (via the S2 subunit) [4]. Previous studies have suggested that the N-terminal domain (NTD) of the S protein has a sugar-binding ability and aids in host receptor binding of PEDV, indicating that the NTD S1 domain may have the potential to be used as a subunit vaccine against PEDV variant strains, due to a potential Ag-specific IgG2a induction [5,6]. In addition, this NTD is a promising candidate antigen for the development of diagnostic tests for PEDV infections [7,8].

In recent years, there has been great interest in the development of immunostimulatory particles as slow antigen release systems [9,10,11,12] which are widely used as platforms that mimic the cell membrane to study protein–protein and protein–lipid interactions, monitor drug delivery, and drug encapsulation [13,14].

Liposome-based immunostimulatory complexes are typically composed of long-chain phospholipids, cholesterol, and saponins. This formulation can generate structures with sizes of approximately 100 nm; these are known for their potential and actual use in targeted drug delivery and vaccine antigen delivery systems, with potent immune-enhancing properties [15,16]. Saponin-based adjuvants show immunostimulatory effects and have been widely used to enhance humoral and cellular immune responses in many species; however, their modes of action are not fully understood [17]. An important saponin in the development of immunostimulant complexes is glycyrrhizic acid (GA), which is derived from licorice root and extracted from the shrub legume *Glycirrhiza glabra* (licorice) root. GA and its derivatives can form intermolecular complexes and micelles containing drug molecules for targeted delivery [18,19]. In addition, formulations based on phospholipids, cholesterol, and GA coupled with a viral antigen generated an increase in immune responses compared to the antigen alone, indicating that GA has promising effects if used within a vaccine antigen delivery system [20,21].

Studies on liposome-based veterinary vaccines have markedly increased, and pig vaccination has become an essential part of pig production; viral vaccines are essential tools for pig producers and veterinarians to manage pig herd health [22]. The ability to induce strong immune responses provided by co-formulated adjuvants, such as liposome-based vaccines, is critical for the development of modern vaccine formulations [23]. This study focused on improving current trends and implementing new technologies for the development of novel porcine virus vaccines. Thus, the aim of this study was to develop and characterize a new lipid NP-based on GA (LNP-GA) by determining its physical properties in terms of size, shape, size distribution, PDI, Z-potential, and transmission electron microscopy (TEM) analysis, and to test its immunogenicity as an adjuvant formulated with a recombinant NTD of the PEDV S protein (rNTD-S-PED) by mice immunization.

## 2. Materials and Methods

### 2.1. Reagents

GA was purchased from Sigma–Aldrich, Burlington, MA, USA, and refrigerated at 4 °C until use; cholesterol and L-α-phosphatidylcholine were purchased from Sigma–Aldrich and stored at −20 °C. Distilled deionized water used throughout the nanoparticle synthesis was filtered using 0.45 µm and then 0.2 µm syringe filters in duplicate. All the other chemical reagents were of analytical grade. A stock solution of GA (10 mg/mL) was prepared by dissolving the acid at a temperature greater than 90 °C in phosphate-buffered saline (1X PBS, pH 7.4), filtered through a 0.45 µm syringe filter, and stored at 4 °C until use. Likewise, solutions of 10 mg/mL cholesterol and L-α-phosphatidylcholine were prepared in chloroform and stored in amber tubes at 4 °C. Tris-HCL (140 mM, pH 7.4), filtered through a 0.45 µm syringe filter, was used as an additional buffer. The solutions were then stored in dark bottles.

### 2.2. Preparation of LNP-GA

LNP-GA was prepared through a lipid film hydration technique [24] by using a procedure similar to the one described by Demana et al. [16], where the ratio of phospholipid:cholesterol:GA was 2:1:2 *v*/*v*, respectively. Briefly, a mixture of 6 mL L-α-phosphatidylcholine (10 mg/mL) and 3 mL cholesterol (10 mg/mL) was evaporated at room temperature overnight. The formed lipid film was hydrated by adding 6 mL of GA solution (10 mg/mL) and 24 mL of Tris-HCl buffer (140 mM, pH 7.4) and mixed using a magnetic stirrer at 300 rpm for approximately 10 min at constant temperature (25 °C) to obtain a final concentration of 5 mg/mL of LNP-GA. A second homogenization was performed at 15,000 rpm for 10 min using an UltraTurrax^®^ (IKA-Werke, Staufen, Germany) digital T-18 rotor-stator homogenizer (IKA-Werke, Staufen, Germany). The final LNP-GA concentration was 5 mg/mL; the homogenized solution was filtered by 0.80 µm, 0.45 µm, and 0.2 µm filters and stored at 4 °C until use.

### 2.3. LNP-GA Characterization by Size Analysis, PDI, and Z-Potential

The particle size and PDI were measured using a DLS NANOSIZER^®^ (Beckman Coulter, Brea, CA, USA) [25]. Briefly, DLS was used to measure average particle diameter and particle diameter distribution in triplicate at a 90° fixed angle for 180 s at 25 °C. The wavelength of the laser light (He/Ne, 10 mW) was set to 678 nm. A digital correlator was used to analyze the scattering intensity data in the unimodal analysis mode. The Z potential was measured by NS ZEN 3600^®^ (Malvern, Worcestershire, UK) at 25 °C in a capillary cell. The electrophoretic mobility of the dispersions was measured and transformed into the Z-potential in triplicate by applying the Smoluchowski approximation at 25 °C in a capillary cell.

### 2.4. Transmission Electron Microscopy (TEM)

The appearance of LNP-GA was observed with a JSM7600-F (Jeol, Akishima, Tokyo) microscope [26]. Briefly, a droplet of the homogenized solution was placed on a copper grid for five minutes. Excess liquid was removed by blotting the grid with filter paper. After the grid had partially dried, a drop of a negative staining solution (2% phosphotungstic acid, *w*/*w*, pH 7.1) was placed on the grid for 5 min. Excess liquid was removed using filter paper, and the grid was dried at room temperature. ImageJ software v. 1.8.0 [27] was used to evaluate the size and dimensions of the nanoparticles.

### 2.5. Production, Expression, and Purification of rNTD-S Recombinant Protein

The open reading frame (ORF) of the PEDV S protein of reference strain PEDV/MEX/MICH/01/2013 (access number: KY828999) was used to design primers (for 5′-CAA GAT GTC ACC AGG TGC TCA GCT A-3′ and rev 5′-GCG CTA CTA AAT ATT AAA CCT CAG AGC C-3′), which hybridize to the NTD domain as reported by Lara et al. [28]. The PCR product was amplified from pJET-NTDS-MICH2013 vector, previously obtained in our work group (data do not show), and the 918 bp fragment was subcloned into Champion™ pET SUMO expression vector (Thermo Fisher Scientific, Waltham, MA, USA) and verified by nucleotide sequencing using Sanger technology at the Biotechnology Institute of Universidad Nacional Autónoma de México (UNAM). Finally, the recombinant plasmid was named pET-SUMO-rNTD-S, and competent cells of *Escherichia coli* (*E. coli*) strain One Shot™ BL21 (DE3) (Invitrogen, Carlsbad, CA, USA) were used to obtain the overproduction strain (BL21-rNTD-S). Cloning and expression were performed according to the procedure described by Lara et al. [29] and García-González et al. [30]. rNTD-S protein was recovered from the inclusion bodies (IB) of 500 mL of induced bacterial cells. The cells were disrupted by mechanical rupture (Gaulin APV Homogenizer Group, Wilmington, MA, USA) in 400 mL of 0.1 M Tris-HCl buffer (50 mM, pH 7.5) for 20 min at 8000 psi. The IB was separated from the mixture via centrifugation and washed with distilled water (5 mL). They were then centrifuged, pelleted, and solubilized in 5% N-lauroylsarcosine sodium salt and 50 mM Tris-HCl pH 7.5 (250 rpm, 12 h, 25 °C). rNTD-S were purified using immobilized-metal affinity chromatography (IMAC) according to the procedure described by Lara et al. [29], it was dialyzed (Tris-HCl 5 mM, pH 8 buffer), quantified with the Bradford method [21], and confirmed by SDS-PAGE and WB before immunization of mice. Detection in WB was performed using anti-6x-His-Tag diluted 1:5000 (Invitrogen, Carlsbad, CA, USA) as primary antibody and a mouse anti-IgG conjugated to horseradish peroxidase (dilution 1:5000) (Sigma-Aldrich, St. Louis, MO, USA) as a secondary antibody.

### 2.6. Structure and Antigenic Epitopes Prediction of NTD-S Protein

Protein structure prediction was visualized and analyzed using the PyMOL software. Antigenic epitope prediction was performed using the method of Kolaskar and Tongaonkar [31], surface probability was determined using the method of Emini [32], and hydrophilicity analysis was performed using the method of Kyte-Doolittle [33]. Molecular modeling of the NTD region of the S1 domain of protein S was performed using the Swiss Model server (Swiss Institute of Bioinformatics, Switzerland) using the trimeric structure of the porcine epidemic diarrhea virus spike glycoprotein (Protein Data Bank accession: 6VV5) as a template [34].

### 2.7. Immunogenicity Evaluation of LNP-GA Coupled to rNTD-S by Mice Immunization and Tested by Indirect Enzyme-Linked Immunosorbent Assay (iELISA)

CF-1 mice (3-week-old) were randomly divided into six groups (*n* = 8 per group). The mice were immunized by a subcutaneous (SC) administration of 200 µL of the formulation into a fold of skin in the neck, and a booster after 2 weeks. All formulations were mixed in a 1:1 mass ratio (5 µg for each component). The immunization and bleeding scheme were as follows: Group 1 was immunized with LNP-GA formulated with recombinant protein rNTD-S (LNP-GA + rNTD-S); Group 2 was immunized with the external reference Matrix-M™ adjuvant (Isconova AB, Uppsala, Sweden) mixed with the rNTD-S (Matrix-M™ + rNTD-S); Group 3 was immunized with GA plus rNTD-S (GA + rNTD-S); Group 4 was immunized with rNTD-S protein alone (rNTD-S + PBS); Group 5was injected with a negative control of LNP-GA alone (LNP-GA + PBS); Group 6 was injected with a blank control of just PBS. For serological analysis, blood samples were collected from the tail vein at days 0, 7, 14, 21, 28, and 35 to test for iELISA using rNTD-S as an antigen to cover the plate. The kinetics of antibody production were measured using iELISA as previously described [21,27]. Statistical analysis was performed using analysis of variance (ANOVA) using NCSS and SigmaPlot statistical programs, with Dunnett’s multiple comparison tests. Statistical *p*-value < 0.05 was regarded as the minimum criterion for statistical significance.

### 2.8. Determination of Pro-Inflammatory Cytokines

The concentrations of the pro-inflammatory cytokines TNF-α and IL-1β were evaluated in the sera of immunized mice from all groups (blank control included). Serum concentrations of both cytokines were evaluated using the commercial ELISA MAXTM Deluxe Set Mouse TNF-α BioLegend kit and the ELISA MAX^TM^ Deluxe set mouse IL-1β BioLegend kit (San Diego, CA, USA). The test was developed based on the indications provided by commercial kits.

All procedures were performed in accordance with Mexican legislation (NOM-062-ZOO-1999; SAGARPA), based on the Guide for the Care and Use of Laboratory Animals, NRC. The experiment was previously approved under a permit from the IACUC (Institutional Animal Care and Use Committee), CENID-SAI, INIFAP. Approval number: CBCURAE-2017/001, approval date: 21 September 2017. The animals were maintained throughout the study period with food and water provided ad libitum and were euthanized by CO_2_ inhalation followed by confirmatory cervical dislocation. 

## 3. Results

### 3.1. Characteristics of LNP-GA

DLS determined the physical properties of the LNP-GA, such as size and PDI. The average particle size of LNP-GA was approximately 200 nm, with a low PDI (<0.2), indicating that LNP-GA formulated with phospholipid:cholesterol:GA in a 2:1:2 ratio using the lipid film hydration technique were relatively monodisperse. As expected, the Z-potential of the LNP-GA was −27.6 mV, indicating high stability of the particles in dispersion. Analysis of the LNP-GA coupled with a recombinant protein rNTD-S in a ratio formulation (1:1) of 5 µg, respectively, showed an average particle size of 347.3 nm and a PDI of 0.648, with a Z-potential value of −21.73 mV; these results show that the particles were homogeneously dispersed and strongly anionic with a relative stability. The GA component had an average particle size of 205.7 nm with a PDI of 1.8, indicating that it was a polydisperse particle. The results are summarized in Table 1. 

### 3.2. Assessment of LNP-GA by TEM

**Figure 1:**
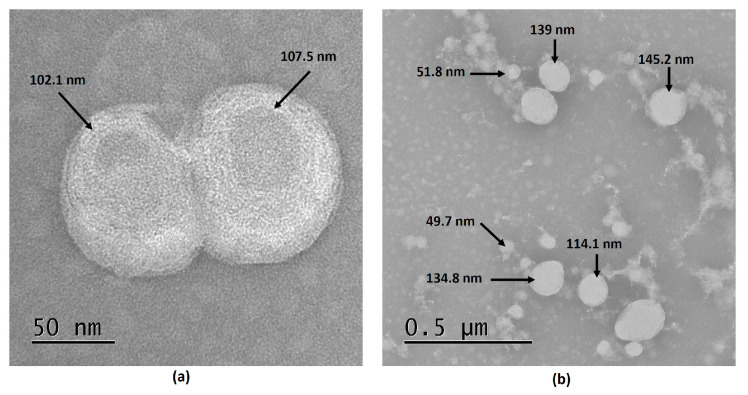
Electron micrography of negative staining preparation of glycyrrhizinic acid-based Lipid Nanoparticle (LNP-GA) (×50,000) (**a**) and LNP-GA (×50,000) coupled with rNTD-S (**b**). The image (**a**) shows spherical bilayer lipid vesicles with an apparently aqueous core. Image (**b**) shows spherical structures with heterogeneity in the size distribution of the population of nanoparticles bound to an electrodense mass associated with the rNTD-S. The evaluation of the micrographs was performed using the ImageJ program.

TEM was used for size analysis and morphological inspection of the LNP-GA. A typical TEM image of LNP-GA is shown in Figure 1a, which shows the diameter distributions at the level of a single particle with an estimated size of approximately 100 nm, as confirmed using the ImageJ program. Transmission electron microscopy analysis identifies a soft spherical bilayer of lipid vesicles, which confirmed the multilamellar structure of LNP-GA with an apparent aqueous core. Shape analysis showed two associated particles with an average size of 100 nm each, suggesting that these structures could be formed during the development of the formulation. TEM analysis of the LNP-GA coupled with a recombinant protein rNTD-S showed a spherical structure with heterogeneity in the size distribution, with the sizes ranging from 49.7 nm to 145.2 nm (Figure 1b).

### 3.3. Production of Recombinant NTD-S Protein (rNTD-S)

**Figure 2:**
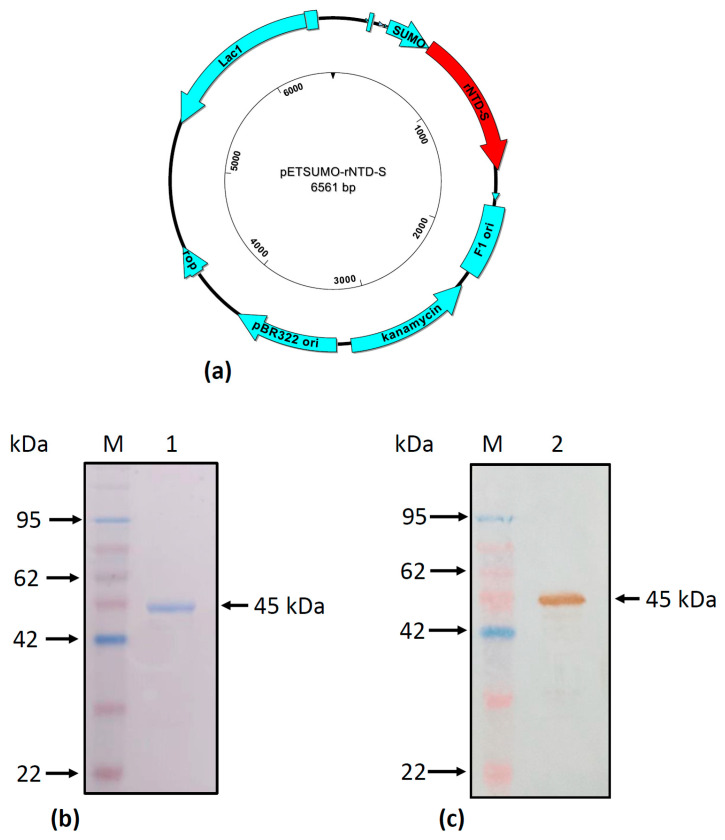
Development and evaluation of PEDV rNTD-S protein expression. Schematic representation of expression vector with rNTD-S coding sequence (**a**), SDS-PAGE gel stained with brilliant blue (Coomassie) (**b**), and Western blot of the rNTD-S protein of porcine epidemic diarrhea virus samples after the purification and dialysis processes (**c**). (M) Marker, (1) Purified rNTD-S [200 ng].

The plasmid pJET-NTD-MICH2013 was subcloned into Champion™ pET SUMO expression vector to express rNTD-S (Figure 2a) protein in *E. coli* strain One Shot™ BL21 (DE3) competent cell (Invitrogen, Carlsbad, CA, USA) using IPTG induction. After 18 h of culturing, the induced cells were collected for analysis using SDS-PAGE, Coomassie staining, and WB. As shown in Figure 2b, black arrow, the rNTD-S protein was identified at the expected molecular weight (45 kDa). Because the protein has a c-myc tag at the C-terminus, its presence can be observed using anti-c-myc; thus, WB analysis was performed (Figure 2c, black arrow). In contrast, no bands were detected in the negative controls. These results confirmed expression of the rNTD-S protein in the *E. coli* vector.

### 3.4. Antigenic Structural Evaluation of NTD-S

**Figure 3:**
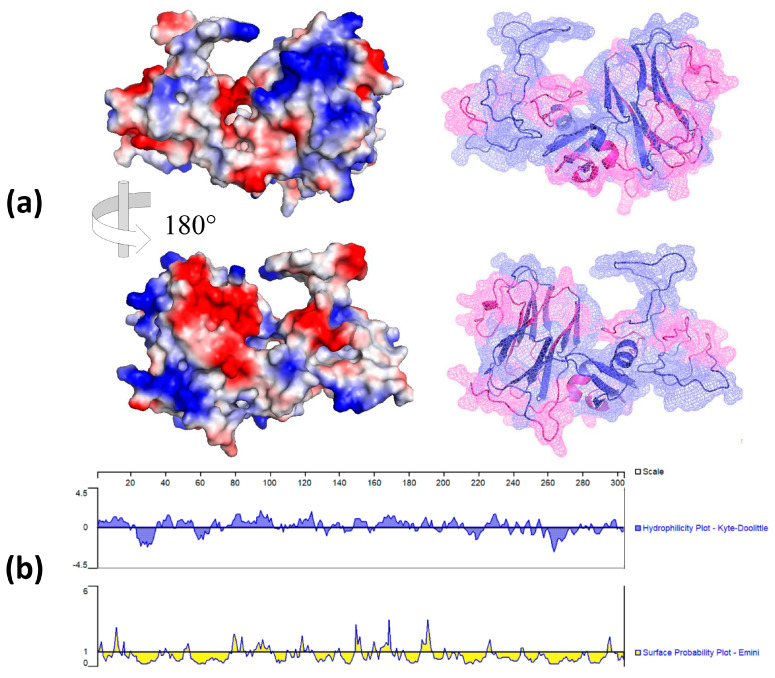
Antigenic structural evaluation of NTD-S protein. (**a**) Molecular model of the NTD region of the S1 domain of the S-glycoprotein vDEP. On the left, the electrostatic surface potential is shown, where the white regions correspond to neutral charges, the red regions correspond to negative charges, the blue regions indicate positive charges, and the right side shows the secondary structure, where antigenic epitopes are highlighted in blue. (**b**) Prediction of hydrophilic regions and surface probabilities.

The secondary structure of the PEDV/MEX/MICH/01/2013 strain was predicted using the spike glycoprotein of vDEP as a template, with 99.67% identity and amino acid coverage from position 11 to 303. The structure (Figure 3a) was visualized using PyMOL, and the electrostatic potential was determined to identify the neutral and charged regions. Antigenic epitope prediction analysis determined 12 sites, which corresponded to large antigenic regions throughout the structure of the recombinant NTD-S1 protein (Figure 3b). Biochemical analysis showed a charge of 2.11, pH of 7, and a titration curve with an isoelectric point (pI) of 7.59.

### 3.5. Antibody Response of Immunized Mice with rNTD-S Coupled to LNP-GA as Adjuvant

**Figure 4:**
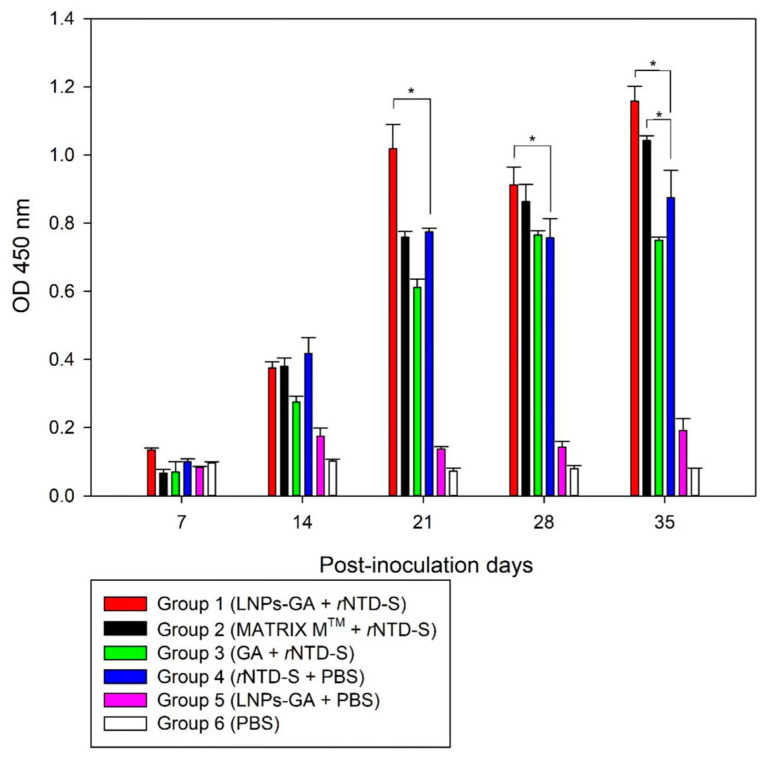
Antibody production in sera from immunized mice with different formulations (ratio 1:1). Graph shows the groups of mice immunized by the different formulations: Group 1: glycyrrhizinic acid-based Lipid Nanoparticle (LNP-GA) coupled with recombinant protein rNTD-S (LNP-GA + rNTD-S); Group 2: reference Matrix-M™ adjuvant coupled with rNTD-S (Matrix-M™ + rNTD-S); Group 3: glycyrrhizinic acid plus rNTD-S (GA + rNTD-S); Grupo 4: rNTD-S protein alone (rNTD-S + PBS); Group 5: control negative LNP-GA alone (LNP-GA + PBS); Group 6: blank control (PBS). *: *p* < 0.05.

The effect of LNP-GA as an adjuvant plus recombinant rNTD-S in vaccinated mice (LNP-GA + rNTD-S, Group 1) was evaluated using data on the kinetics of antibody production by iELISA. An increased level of antibody production in anti-rNTD-S sera was detected from day 14 post-inoculation compared with that in mice immunized with the reference Matrix-M™ adjuvant (Isconova AB, Uppsala, Sweden) formulated with rNTD-S (Matrix-M™ + rNTD-S, Group 2). In particular, LNP-GA as an adjuvant showed a higher immune response in mice on day 21 compared to all other groups, and significant differences were observed with respect to the immune response of negative controls (Groups 5 and 6) from day 21 to day 35 (*p* < 0.05), indicating the efficiency of this formulation (Figure 4). Conversely, a slow immune response was observed in mice immunized with rNTD-S formulated with GA (group 3), suggesting possible capture of the recombinant protein in the release process.

Mice immunized with rNTD-S alone (rNTD-S + PBS, group 4) presented high levels of antibody production from day 14 post-inoculation, indicating that the purified recombinant protein may be used as a good vaccine candidate against PEDV, where the immune response was improved using the new formulation as adjuvant. In addition, no adverse reactions were observed at the injection site or in the overall health of the mice after vaccination.

### 3.6. Potential Anti-Inflammatory Effect Study

**Figure 5:**
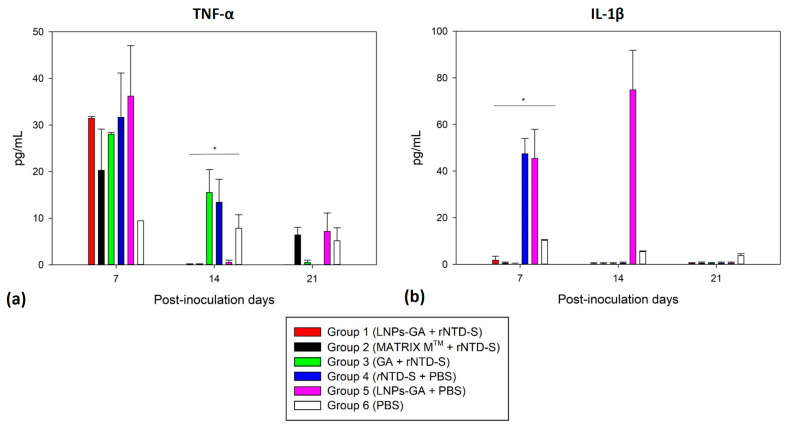
Determination of pro-inflammatory TNF-α (**a**) and IL-1β cytokines (**b**). Graphs show the serum concentrations of cytokines in immunized mice (blank included). *: *p* < 0.05.

The effect of TNF-α and IL-1β concentrations in serum samples from the target group (blank immunized with PBS) was minimal, and the data were used as a reference for the other assessed groups. The results in Figure 5a,b demonstrate that the group inoculated with LNP-GA coupled rNTD-S had reduced levels of TNF-α from day 14 and IL-1β from day 7, compared to the protein alone and the target groups. Then, in immunized mice, the LNP-GA complex as an adjuvant and rNTD-S did not generate a substantial first pro-inflammatory cytokine response.

## 4. Discussion

Significant effort has recently been made to develop LNPs as efficient delivery systems for different antigens [35], as well as and to develop techniques that enable the prevention or treatment of infections by boosting the immune response against the target pathogens, which has led to the evolution of vaccines. For example, significant progress in the evaluation of liposomal nanoparticles (LNP) as vaccine delivery systems or immunogenic mechanisms has recently been made, as evidenced by the development of effective LNP-based vaccines against COVID-19 [22]. In the present study, an immunostimulant complex of LNP-GA was developed as an efficient adjuvant and formulated using the recombinant protein rNTD-S of PEDV. Characterization of the immune response in immunized CF-1 mice revealed superior humoral immunity from day 27 post-immunization compared with the other groups tested with different protein mixtures.

The lipid film hydration technique was used to carry out the formulation of LNP-GA and proved to be fast and efficient; with results obtained comparable to the ones obtained by Demana [16]. LNP-GA was easily prepared and showed efficient humoral responses when coupled with a recombinant antigen of PEDV, indicating that the ratio used in the formulation of LNP (phospholipid:cholesterol:GA:2:1:2) was ideal, especially the cholesterol ratio, because moderate amounts of cholesterol can increase the ordered arrangement of lipid membranes and their stability, consequently favoring the penetration of drugs into the lipid shell [29].

Several studies have shown that due to its physicochemical characteristics, GA is inserted within the lipid nanoparticle framework, generating structures with expected sizes around 200 ± 50 nm [18,35]. In this respect, Brewer [36] and Mann [37] analyzed particles with sizes between 560 and 225 nm and showed that populations with an average diameter of 250 nm induced significantly higher IgG2a and IFN-γ levels post-antigen and mitogen stimulation in lymph node cultures. Therefore, our results on the physical characteristics (i.e., size, PDI, and Z-potential) of LNP-GA showed an average size of 211.5 nm, suggesting its capacity and efficacy to generate good immunogenic effects. In addition, the LNP-GA formulation produced structures with a PDI of approximately 0.283, which shows a suspension size distribution consistent with previous publications [38,39]. Furthermore, Z-potential analysis, which indicates the charge acquired by NPs in a dispersed medium resulting from the surface charge, concentration, and types of components in solution, was used as a measure of stability in solution [40]. The value of −27.6 mV from the LNP-GA formulation, suggests that these particles were sufficiently dispersed, and it was unlikely that they undergo flocculation or form aggregates with each other. The Z value (−27.6 mV) also confirmed the stability of the size and charge of the particles in aqueous solution because it has been reported that particles with Z-potentials close to or greater than ±30 mV are stable in size and charge [41]. Interestingly, the particle size analysis as well as dispersion assessment of GA indicates that in absence of lipids, GA alone could easily form aggregates [18]. However, our analysis of the physical properties of the LNPs using GA in the formulation revealed that the inclusion of components such as cholesterol and phospholipids formed a more stable complex, resulting in nanoparticles of more homogeneous size and dispersion.

We suggest that the rNTD-S1 protein interacts with the nanoparticles via adsorption, due to its pI of 7.59 and the Tris-HCl buffer (140 mM, pH 7.4) in which the nanoparticles were synthesized, since previous reports showed that at pH values closer to the pI the interactions between the proteins themselves are reduced, thus increasing the adsorption of the proteins towards the nanoparticles [23]. In addition, the charge of the NTD-S1 protein is slightly positive (2.11 mV), which favors the interactions with nanoparticles composed of phospholipids, which are negatively charged by nature and whose interaction takes place in the hydrophilic region of the lipid aggregates [42]. As expected, the data obtained for the LNP-GA formulated with rNTD-S showed an increase in their size and PDI (347.3 nm and 0.648, respectively), indicating the presence of subpopulations of LNPs with a heterogeneous size distribution.

The effect of LNP-GA as an adjuvant formulated with rNTD-S on the humoral immune response in vaccinated mice was significantly greater than the negative control group (*p* < 0.05). The highest immune response was also observed from day 21 post-inoculation until day 35 compared with mice immunized with rNTD-S alone, indicating that the bioavailability of the recombinant antigen increased after coupling with LNP-GA and despite the modifications in the final mixture of LNP-GA coupled to rNTD-S, the specific antibodies recognized the recombinant protein in ELISA. In conclusion, recombinant protein retained the structure necessary to produce an immune response. These results agree with those described by Zhao [20], who also successfully used GA to induce an efficient humoral immune response in chickens against Newcastle disease after GA was encapsulated in liposomes and coupled to the Newcastle disease vaccine.

As expected, the humoral immune response induced by LNP-GA plus rNTD-S was comparable to that produced with the reference Matrix-MTM adjuvant [40,43], indicating that the LNP-GA formulation coupled with the recombinant protein as an adjuvant could be an important carrier system in vaccine development.

In addition, the kinetics of the antibody response observed in immunized mice with rNTD-S alone enhanced immunity after 14 days post-inoculation, confirming the antigenicity of the NTD of the PEDV S protein. This finding is consistent with previous reports [5], in which the NTD was shown to induce antigen-specific immune responses in both the systemic and mucosal immune compartments when administered orally. Furthermore, the NTD of the PEDV S1 domain has been investigated as a binding sugar and putative co-receptor for PEDV [44] and positive sera pigs tested by indirect enzyme-linked immunosorbent assays making use of truncated recombinant S1 protein were also correlated with virus neutralization tests [7]. Therefore, the recombinant protein rNTD-S could be an efficient antigen for the development of a potential candidate vaccine or diagnostic system for PEDV. This aspect is important because research on next-generation vaccines, such as RNA and DNA-based vaccines as well as subunits, and viral-vector approaches, are critical for the prevention of future outbreaks of emerging coronavirus diseases, such as PEDV [44,45,46].

In this study, analysis of the TNF-α and IL-1β cytokines were found in lower amounts in sera from mice vaccinated with the LNP complex, indicating that LNP-GA did not appear to generate any harmful inflammatory response in mice; however, PEDV infection in pigs increases the production of pro-inflammatory cytokines (TNF-α, IL-6, IL-8, IL-12, IL-17, IFNα, and IL-22) and in pathological processes, these can become markers in the evolution of the disease, such as in PED [3]. Previous studies have shown that GA has inhibitory effects on proinflammatory cytokines such as TNF-α and IL-1β [47]. Therefore, the results indicate that the decrease in the levels of these two cytokines can be attributed to the presence of GA in LNP. Interestingly, Group 3 showed elevated levels of these cytokines. As previous studies have shown that GA is highly soluble in aqueous media [48], this would probably indicate that GA alone is rapidly distributed in the bloodstream, decreasing its selectivity in target cells, which could indicate that GA-containing LNPs have a longer duration and selectivity in the bloodstream. In addition, a recent study showed that LNP-GA can increase IL-10 levels in the serum of immunized mice [21], indicating that LNP-GA could play an anti-inflammatory role, which could be vital in the process of immunization and control of PEDV infection. Further studies are necessary to elucidate the mechanisms underlying this effect.

In this study, we developed rNTD-S, a recombinant antigen that was demonstrated to be efficacious in producing antibodies in a mouse model when combined with LNP-GA as adjuvant. The lipid nanocarriers system developed with GA not only improved antibody levels but also reduced pro-inflammatory cytokines in serum. Finally, due to the immunogenic potential of the LNP-GA–rNTD-S complex, its potential application for preventing and managing swine epidemic diarrhea disease may be studied further in pig trials. Vaccination has become an important part of pig farming, and viral vaccines are important tools for pig farmers and veterinarians to use to maintain the health of their herds [22].

## 5. Conclusions

All of these findings support the use of LNP-GA as a lipid-based nano-carrier system which could potentially be used as an adjuvant for the development of subunit vaccines based on recombinant proteins, such as rNTD-S. The LNP-GA coupled to rNTD-S protein will be examined further in pig trials for its suitability for preventing and controlling swine epidemic diarrhea disease.

## Figures and Tables

**Table 1:** Mean particle size, polydispersion index, and lipid-nanoparticles stability assessment (Z-potential). The values were measured in a Dynamic Light Scattering NANOSIZER^®^ (Beckman Coulter, Brea, CA, USA).

Sample	Mean Particle Size (nm) *	Polydispersion (PDI) *	Z-Potential (mV) **
Recombinant N-terminal domain of the PEDV spike protein (*r*NTD-S)	2150.3	1.715	−9.13 ± 4
Glycyrrhizinic acid (GA)	205.7	1.8	−16.29 ± 7.32
Glycyrrhizinic acid-based Lipid Nanoparticle (LNPs-GA)	211.5	0.283	−27.6 ± 9.19
Glycyrrhizinic acid-based Lipid Nanoparticle (LNPs-GA) plus *r*NTD-S	347.3	0.648	−21.73 ± 8.41

* Reported as mean; n = 3. ** Reported as mean ± standard deviation; *n* = 3.

## Data Availability

The data underlying this article will be shared on reasonable request to the corresponding author.
